# Analysis of the Microbiota of Milk from Holstein–Friesian Dairy Cows Fed a Microbial Supplement

**DOI:** 10.3390/ani15142124

**Published:** 2025-07-18

**Authors:** Bronwyn E. Campbell, Mohammad Mahmudul Hassan, Timothy Olchowy, Shahab Ranjbar, Martin Soust, Orlando Ramirez-Garzon, Rafat Al Jassim, Robert J. Moore, John I. Alawneh

**Affiliations:** 1 School of Science, RMIT University, Melbourne, VIC 3083, Australia; rob.moore@rmit.edu.au; 2 School of Veterinary Science, The University of Queensland, Gatton, QLD 4343, Australias.ranjbar@uq.edu.au (S.R.); 3 Faculty of Veterinary Medicine, University of Calgary, Calgary, AB T3R 1J3, Canada; timothy.olchowy@ucalgary.ca; 4 Terragen Biotech Pty Ltd., Coolum Beach, QLD 4573, Australia; msoust@me.com (M.S.); o.ramirez@uq.edu.au (O.R.-G.); 5 Queensland Alliance for Agriculture and Food Innovation, Brisbane, QLD 4072, Australia; r.aljassim@uq.edu.au; 6 Plant Biosecurity and Product Integrity, Biosecurity Queensland, Department of Primary Industries, Brisbane, QLD 4001, Australia

**Keywords:** direct-fed microbial, DFM, milk microbiota, dairy cows, bacterial diversity, productivity

## Abstract

Previous studies of direct-fed microbial (DFM) supplements showed variable effects on the microbiota and physiology of dairy cows. The main aims of this study were to investigate the milk microbiota of cows supplemented with a lactobacilli-based DFM compared to untreated cows; describe the changes; and quantify the association between the taxa and cow productivity. The study followed seventy-five Holstein–Friesian dairy cows supplemented with a DFM over 16 months compared to seventy-five non-supplemented cows. Twenty-five cows from each group were sampled for microbiota analysis. The top taxa significantly associated with the variables were as follows: Age (*Mammaliicoccus*_319276, *Turicibacter*), milk production (*Turicibacter*, *Bifidobacterium*_388775), DIM (*Stenotrophomonas*_A_615274, *Pedobacter*_887417), milk fat percentage (*Pseudomonas*_E_647464, *Lactobacillus*), calendar month (*Jeotgalicoccus*_A_310962, *Planococcus*), milk protein percentage (*Tistrella*, *Pseudomonas_E_650325*), experimental group (*Enterococcus*_B, *Aeromonas*), SCC (*Paenochrobactrum*, *Pseudochrobactrum*), and trimester of pregnancy (*Dyadobacter*_906144, VFJN01 (Acidimicrobiales)). These were identified using multivariable analysis. Twenty-six genera were associated with the differences between experimental groups, including *Pseudomonas*, *Lactococcus* and *Staphylococcus*. Microbial taxa that changed in relative abundance over time included *Atopostipes*, *Brevibacterium* and *Succinivibrio.* Many of these genera were also part of the core microbiota. Supplementation with the DFM significantly altered the milk microbiota composition in the dairy cows, highlighting the impact of long-term DFM supplementation on microbial communities.

## Simple Summary

This study reports the investigation of the effects of a lactobacilli-based direct-fed microbial (DFM) supplement on milk microbiota and production of dairy cows over a 16-month period. Significant differences between cows receiving the supplement compared to those that did not were identified. Supplementation with a DFM can improve dairy cow productivity, including increasing the quality and quantity of milk produced. An understanding of how these supplements influence the existing microbiota is critical in optimizing their use, formulation, and effectiveness and for enhancing animal health and welfare. This research highlights the potential of using DFMs to improve the efficiency of the dairy industry.

## 1. Introduction

Direct-fed microbial (DFM) supplements are used in cattle, aiming to improve health and productivity. The findings of DFM supplementation trials have been inconsistent, likely due to the variability in dosage, the frequency of dosage, the species or strains used, and host factors, such as gut and intestinal conditions, and the microbiota already present in the animals [1,2,3]. Host factors can also include the age of the animal, the current health status of the animal and, in cattle, the stage of lactation and the number of lactations the cow has had. The most significant effects of DFM supplementation were reported in situations where the calves or cows were unwell, with DFMs used to ameliorate diarrhea in calves and ruminal acidosis in cows [1,4,5]. Xu et al. [6] used a lactobacilli-based DFM to improve milk production and quality and found improved milk production of up to 9 kg/day on day 30 of supplementation. In a comprehensive review, El Jeni et al. [3] identified seven ways these DFMs may exert their effects on dairy cows, usually via influencing the ruminal environment, immune system, and existing microbiota. Previous studies have shown that supplementation with a lactobacilli-based DFM can favor growth of beneficial bacteria whilst suppressing growth of pathogenic bacteria. Beneficial species include those associated with digestion of plant material and production of volatile fatty acids, such as those belonging to the genera *Prevotella*, *Ruminococcus*, *Bifidobacterium*, *Faecalibacterium* and *Roseburia.* Improvements in feed conversion and energy production could improve milk production. The lactobacilli themselves can also stabilize ruminal pH, by promoting lactic acid utilizing bacteria such as *Megasphaera* sp., important for preventing ruminal acidosis [7,8,9]. A stable microbiota can also reduce the presence of pathogens such as those associated with mastitis [3,9].

Specific microbial genera in milk, including *Acinetobacter*, *Pseudomonas*, *Stenotrophomonas*, *Lactococcus* and *Chryseobacterium*, consistently exhibit higher abundances than other genera regardless of geographical area, season, or lactation stage [10,11,12]. In fact, taxa in the phyla *Pseudomonadota*, *Bacillota*, *Bacteroidota* and *Actinomycetota* account for more than 95% of the total relative abundance, consistently prevailing across various conditions and samples [12].

Obtaining a milk sample under aseptic conditions is a major challenge to milk microbiota studies. The milk sample is very easily contaminated by microbiota from the environment, including bedding, teat skin, teat canal, and feces [13,14,15,16]. There are significant differences in microbiota composition across the teat skin, teat canal, and udder [17]. The teat skin harbors a diverse microbial community primarily consisting of environmental bacteria, while the teat canal has a more specialized microbiota that can act as a barrier against mastitis-associated bacterial species [18]. The udder itself has its own microbiota, with specific beneficial bacteria, such as non-aureus *Staphylococcus chromogenes*, that play a role in maintaining udder health and preventing infections [11,13,14]. Milk taken aseptically directly from the mammary gland has few viable bacteria [16]. Analysis of the microbiota by culture-independent methods, such as next-generation sequencing technologies, has opened new pathways for investigating the milk microbiota. Like ruminal studies, researchers can now investigate the effects of DFM supplementation on milk production. A ‘core’ microbiota can be identified, and different taxa can be directly related to the productivity of the cows.

The main aim of this study was to characterise the potential effects of supplementation with a well-characterised and standardised lactobacilli-based DFM on the milk microbiota of dairy cows, compared to untreated controls. Building on evidence of DFM-induced changes in rumen and intestinal microbiota and the gut-mammary axis connection, we sought to describe alterations in the milk microbiota and to identify specific taxa associated with factors such as age, average daily milk production, days in milk, milk fat and protein content, calendar month, somatic cell counts, and pregnancy trimester.

## 2. Materials and Methods

### 2.1. Direct Fed Microbial

The DFM formulation used, MYLO^®^ (Terragen Pty Ltd., Coolum Beach, QLD, Australia), contained live *Lacticaseibacillus casei* Lz26, *Lentilactobacillus buchneri* Lb23, and *Lacticaseibacillus paracasei* T9 at around 3.5 × 10^9^ cfu/mL each [19].

### 2.2. Cows and Sampling

The study set-up, sampling, and metadata measurements are described in detail by Ramirez-Garzon et al. [19]. In brief, the study animals comprised of first and second lactation Holstein–Friesian dairy cows aged 2–4 years at the time of the study. Cows were selected based on parity and days in milk (DIM). Both primiparous and multiparous cows were included in the study, initial DIM at 87–247 days and average milk production per day of 18–32 L. The average liveweight of the cows used in the study was 590 ± 67 kg.

Seventy-five cows (SUP group) were supplemented with the DFM (10 mL/cow/day), added to the partial mixed ration (PMR) on a feed pad, and given each morning (6 am). Feeding consisted of PMR during the day within a dry lot and pasture grazing at night. Mixed ration consisted of maize or barley silage, lucerne hay, soybean silage, canola meal and barley or wheat grain. The barley or wheat grain (1.5 kg as fed) was fed to the cows twice a day. Pasture consisted of ryegrass in the winter and kikuyu in the summer (up to 6 kg dry matter [19]. A further seventy-five cows were used as non-supplemented control cows (CON), receiving the same PMR without DFM. Cows were retained in separate yards during the day and at nighttime were allowed to graze in separate paddocks. Over the course of the study some cows were dried off in February/April 2022. Cows that were suspected of, or diagnosed with, mastitis were segregated and received the appropriate treatment, and the details of cow ID, dates and duration of infection and treatment regime noted.

Milk samples, twenty-five each chosen at random from the seventy-five in the CON and SUP groups, were taken at approximately two-month intervals, for 16 months, for eight-time points. Cows were milked twice daily, at 4 am and 3 pm, with samples for microbiota analysis taken before the routine afternoon milking. This was performed as the DFM was provided to the cows at 6 am after the first milking and to also give the DFM time to exert its effects on the microbiota. The same twenty-five cows sub-sampled from the seventy-five from each experimental group were sampled across time for the microbiota analysis. Before milk collection, each teat was thoroughly cleaned using a cotton pad soaked in 70% ethanol (Chem-Supply, Adelaide, Australia). Each udder quarter was hand-stripped and individual milk samples collected into sterile 50 mL polypropylene tubes and placed immediately on ice. Samples were stored at 4 °C and, within 5 h of sampling, 4 mL was placed into a sterile 5 mL PP flat bottomed sample tube (Interpath, Melbourne, Australia) and labelled with the cow’s ID, date and group. Samples were stored at −20 °C before being shipped on dry ice to the RMIT laboratory for microbiota analysis.

Every 6–8 weeks, composite milk samples were collected as part of the herd’s udder health and mastitis control management strategy for milk fat (FatPerc) and protein (ProtPerc) and somatic cell count (SCC) analyses. The milk was collected from the on-farm automated milk sampling meters as detailed above, placed into vials containing the preservative Bronopol (Novachem, Melbourne, Australia) and shipped to Dairy Express Herd Recording Service (Armidale, NSW, Australia) for analysis. All cows, except for the afternoon sampling for microbiota, were routinely pre-dipped prior to milking.

Sampling was different for microbiota studies compared to SCC, FatPerc and ProtPerc analysis for two main reasons. Firstly, to prevent possible in-line contamination of microbiota samples if they were taken from the automated milk sampling meters. Direct sampling of cows for microbiota analyses was considered better. Secondly, to have the most ideal storage of milk for analyses. For DNA analysis the samples need to be frozen to preserve the DNA in the best possible form. Also, Bronopol, used for milk preservation, can produce formaldehyde which would require additional processing before PCR and sequencing could occur. Whilst frozen milk can be used for SCC, FatPerc and ProtPerc it is not ideal and can result in lower SCC counts detected.

### 2.3. DNA Extraction

One mL of milk was centrifuged at 16,000× *g* for 15 min at 4 °C. The cream plug at the top was removed using individual sterile inoculation loops and then discarded. The supernatant was removed and replaced with 1 mL of ice-cold 0.85% sterile saline. The pellet was re-suspended by repeated pipetting, and the sample was centrifuged at 16,000× *g* for 10 min at 4 °C. Any remaining cream and the supernatant were removed. The pellet was resuspended in lysis buffer with proteinase K from the Maxwell^®^ RSC Fecal Microbiome DNA Kit (Promega, Madison, WI, USA) and transferred to a Matrix E tube for bead-beating (MP Biomedicals, Santa Ana, CA, USA). Samples were subjected to bead-beating for 2 cycles of 1 min at 4 m/s with a 5-min break between cycles. Tubes were incubated at 95 °C for 5 min, vortexed for 1 min, and then incubated at 56 °C for 5 min. Samples, including a negative control, which included the lysis and binding buffers but no sample, were loaded into the cartridges of the kit and extracted automatically on a Maxwell^®^ RSC instrument (Promega, Madison, WI, USA). The quality and quantity of DNA were assessed using a NanoDrop™ spectrophotometer (Thermo Fisher Scientific, Waltham, MA, USA) and Qubit HS DNA assay (Thermo Fisher Scientific, Waltham, MA, USA), respectively, according to the manufacturer’s instructions.

### 2.4. 16S rRNA Gene PCR and Sequencing

The V3–V4 region of the 16S ribosomal RNA gene was amplified and indexed in two rounds of PCR, sequenced, and analyzed according to the protocol of Campbell and Van [20]. In brief, degenerate primers capable of amplifying all bacteria were used to amplify 16S rRNA genes in the first round of PCR. BacF: 5′ GTCTCGTGGGCTCGGAGATGTGTATAAGAGACAGGGACTACHVGGGTWTCTAAT 3′ and BacR 5′ TCGTCGGCAGCGTCAGATGTGTATAAGAGACAGCCTACGGGAGGCAGCAG 3′ [2,21,22]. PCR reactions included: 1 µL DNA, 0.5 µL dimethyl sulfoxide (DMSO), 5 µL of KAPA HiFi HotStart ReadyMix (2×, Roche), 0.3 µL BacF (10 mM), 0.3 µL BacR (10 mM), 2.9 µL water. Thermocycling conditions were 95 °C/5 min: 30 cycles of 98 °C/20 s; 55 °C/15 s; 72 °C/1 min. The negative control reactions replaced the DNA with water.

Template from the first PCR, including the negative controls, was used for the indexing PCR with Nextera XT Index 1 plate forward indexes (Illumina, San Diego, CA, USA) and a single reverse index per plate (R97, R98, R99, Illumina, San Diego, CA, USA). The PCR reaction mixture included 1.5 µL of round 1 PCR product, 0.5 µL DMSO, 5 µL of KAPA HiFi HotStart ReadyMix, 0.5 µL forward index (10 mM), 0.5 µL reverse index (10 mM), 2 µL water. Thermocycling conditions were 95 °C/5 min: 10 cycles of 98 °C/20 s; 55 °C/15 s; 72 °C/10 min.

All PCR reactions, for both rounds of PCR, were run on 2% agarose gels and stained with 1 × GelRed (Biotium, Fremont, CA, USA) to ensure that amplification was specific, amplicons were the correct size, and that no amplification occurred in the negative controls. Library pooling, including negative controls even though no amplicons were visible, and cleanup followed the Illumina 16S Metagenomic Sequencing Library preparation document (#15044223). Amplicons were sequenced on a MiSeq (Illumina, San Diego, CA, USA) using the MiSeq Reagent Kit (v3, 600 cycle, 300 bp reads), at a ratio of 90% 16S amplicon and 10% PhiX control library (PhiX Control Kit v3, Illumina, San Diego, CA, USA) and a final concentration of 7–8 pM.

### 2.5. Bioinformatic and Statistical Analyses

The goal of the 16S amplicon sequencing was to generate a minimum of 30,000 good-quality reads per sample. The quality of sequences and an assessment of the need for trimming was performed using FastQC (v0.12.1, http://www.bioinformatics.babraham.ac.uk/projects/fastqc/, accessed on 1 September 2024). QIIME2 was used to demultiplex the sequences, and the DADA2 plugin was used to denoise and trim sequences [23,24]. Amplicon sequence variants were filtered by feature and sample and then summarized to give final representative sequences. Amplified sequence variants (ASV) were classified into taxa by comparison with the GreenGenes2 database (v2, https://greengenes2.ucsd.edu/, accessed on 1 September 2024; [25]) and, in combination with metadata information, summarized into a feature table. ASVs were then aligned, to group sequences with high homology, masked to remove ambiguous sequences, and a phylogenetic tree constructed using Fasttree in QIIME2. For further analysis, using MicrobiomeAnalyst (MA) (https://www.microbiomeanalyst.ca, accessed on 1 September 2024), four files were exported from QIIME2: feature-table.csv, taxonomy.csv, metadata.csv, and the phylogenetic tree in Newick format (.nwk). The first analysis in MA was to plot sequence sample size against species richness to ensure that adequate sequencing had been performed to detect the taxa present. Data was then investigated by taxonomic classification, with MA able to display any taxonomic level over time and supplementation graphically. Alpha-diversity analysis was performed to investigate whether there were significant within-experimental group or within-time-point differences in the richness and evenness of genera. The indices used in the analysis were observed, Chao1 [26] and Shannon [27]. Beta-diversity, via non-metric multidimensional scaling (NMDS; [28]) and principal coordinate analysis (PCoA; [29]), was used to determine whether microbial diversity was significantly different between CON and SUP cows and between time points. Core microbiota analysis identified taxa that were common to samples within each experimental group. Linear discriminant analysis effect size (LEfSe) was used to determine which taxa were driving any differences seen in CON and SUP cows over time. For all statistical analyses, *p* values ≤0.05 were deemed significant.

The main dataset comprised experimental group as a categorical variable (supplementation with (SUP) and without DFM (CON)), calendar month of sampling (as a categorical variable; month), cow age (as a continuous variable in years), DIM as a categorical variable (arbitrarily categorized into dry period [0 DIM], early lactation [9–99 DIM], Mid-lactation [100–200 DIM] and Late lactation [201–536 DIM], no milk data were available for 1–8 DIM), trimester of pregnancy (trimester) as a categorical variable (categorized as trimester 1 [1–3 months], trimester 2 [3–6 months] and trimester 3 [> 6 months]) and cow’s average milk per day (l; Average), log somatic cell count (SCC) (SCC × 1000 cells/mL), percentage of fat (FatPerc) and protein (ProtPerc) in the milk. The categorical variables were chosen to provide the ability to measure the effect of any changes in the microbiota due to the DFM on the health (SCC) and milk production (average) and quality (SCC, FatPerc, ProtPerc) of the cows, whilst accounting for the effects of time (sampling time and cow age) and reproductive status (DIM, trimester, lactation).

Multivariable analysis (MaAsLin2 v1.15.1; [30]) was then used to quantify the association between bacterial taxa and cow data. The model used total sum scaling normalization (TSS) and arc-sine square root transformation of the data to account for instances of zero abundance. *p*-values were then adjusted using the Benjamini-Hochberg false-discovery method at a threshold of 0.2 [31]. Initial analysis was unadjusted, with multiple bivariable models using experimental group with individual explanatory variables. Following interpretation of these outputs, a larger analysis was performed using all variables together. For the categorical variables in the analyses, the following categories were used as references: Experimental group (CON), Month (Sep21), and Trimester (First). Graphical display of the multivariable analysis was performed using ggplot2 (v3.5.1 [32]; in Rstudio (1 September 2024, Build 394; [33]). Some studies have correlated the ratios of Bacillota to Bacteroidota with the proportion of fat in the milk [34,35,36,37,38], so correlation analysis was performed in this study to determine whether this association could be detected in all data together. Correlation analysis was also performed to determine whether there was a significant relationship of this same ratio in the ruminal microbiota, reported in Campbell et al. [39] with the milk microbiota reported herein. Ramirez-Garzon et al. [19] provides the full analysis of the physiological effects of the DFM on SCC, average, FatPerc and ProtPerc. The variation in SCC due to the DFM was assessed using an F-test, comparing the variance within and between CON and SUP groups. The effects of the DFM on average milk production was tested using a *t*-test over each sampling time of the study and overall.

## 3. Results

A total of 18,171,114 16S rRNA gene amplicon sequences were produced, with a pre-filtering average of 60,169 reads per sample. Following stringent filtering and quality control, the average number of reads per sample was 31,857. Plotting of the sequence coverage against amplified sequence variant richness indicated that there were sufficient sequences to give a comprehensive coverage of the variants present.

Taxonomic classification, using GreenGenes2, classified 99%, 98%, 72%, and 21% of the milk microbiota to the levels of phylum, family, genus, and species, respectively. The bacterial phyla present in milk in both CON and SUP cows over time are shown in Supplementary Figure S1 and the data for classification to phylum, family and genus are provided in Supplementary File S2. A total of 503 genera within 228 families were identified. The most prevalent microbial taxa found in the present study, and other milk microbiota studies, within the phylum Bacillota were *Staphylococcus*, *Streptococcus*, *Lachnospiraceae*, *Ruminococcaceae*, *Enterococcus*, *Clostridiales* and *Aerococcus*. Within the phylum Bacteroidota were *Prevotella*, *Bacteroidales*, *Flavobacteriaceae*, and *Sphingobacterium*. The phylum Actinomycetota included *Corynebacterium*, *Bifidobacterium*, and *Propionibacterium*. Finally, the phylum Pseudomonadota included *Acinetobacter*, *Pseudomonas*, and *Stenotrophomonas* [14,40]. Oikonomou et al. [40] also indicated the presence of *Romboustia*, *Turicibacter*, and *Dietzia*. Genera important for food technology applications, including for the production and maturation of cheese, include *Lactococcus*, *Lactobacillus*, *Streptococcus*, *Leuconostoc*, *Enterococcus*, and *Propionibacterium*. The spoilage bacteria are *Pseudomonas*, *Acinetobacter*, *Chryseobacterium*, and *Clostridium*. Species in some genera (*Listeria*, *Staphylococcus*, *Escherichia coli*, *Campylobacter*, *Mycobacterium*) are responsible for disease or illness [41]. According to the review by Quigley et al. [41], those most prevalent in milk were *Lactobacillus*, *Staphylococcus*, *Lactococcus*, *Leuconostoc*, *Streptococcus*, *Corynebacterium*, *Weissella*, *Propionibacterium*, *Pseudomonas*, *Sphingomonas*, *Ralstonia* and *Serratia*. In the present study, no *Propionibacterium*, *Ralstonia*, or *Leuconostoc*, were found in the milk samples, and *Streptococcus*, *Sphingomonas*, and *Serratia* were low in prevalence. Of the species deemed less prevalent but commonly detected were *Acinetobacter* and *Citrobacter*, the former genus was the most prevalent genus detected in the present study, and the latter was low in prevalence. Of the thirteen genera found occasionally, the present study detected low levels of *Bifidobacterium* (0.3–0.4%), *Enterococcus* (0.13–0.6%), *Lactobacillus* (0–0.01%), *Rothia* (0.02–0.04%) and *Prevotella* (0.01–0.02%). The genus *Stenotrophomonas* (3.7–6.4%), found occasionally in other studies, was present as *Stenotrophomonas*_A_615274 at relatively high prevalence. Many taxa in milk analyzed in the present study were part of the core microbiota of both CON and SUP cows. The most prevalent taxa in the core microbiotas were *Acinetobacter*, *Pseudomonas*, and *Stenotrophomonas*, all Pseudomonadota associated with milk spoilage. They have, however, also been associated with milk from healthy udder quarters [42,43]. Those associated with food technology applications (*Lactococcus)* and disease (*Staphylococcus*, *Klebsiella*) were also part of both core microbiota. Of the top twenty most prevalent taxa of the core microbiota, those associated with spoilage (*Acinetobacter*, *Chryseobacterium*, *Clostridium*, *Pseudomonas*) were equally prevalent in both CON and SUP cows or at slightly higher prevalences in SUP cows.

**Figure 1:**
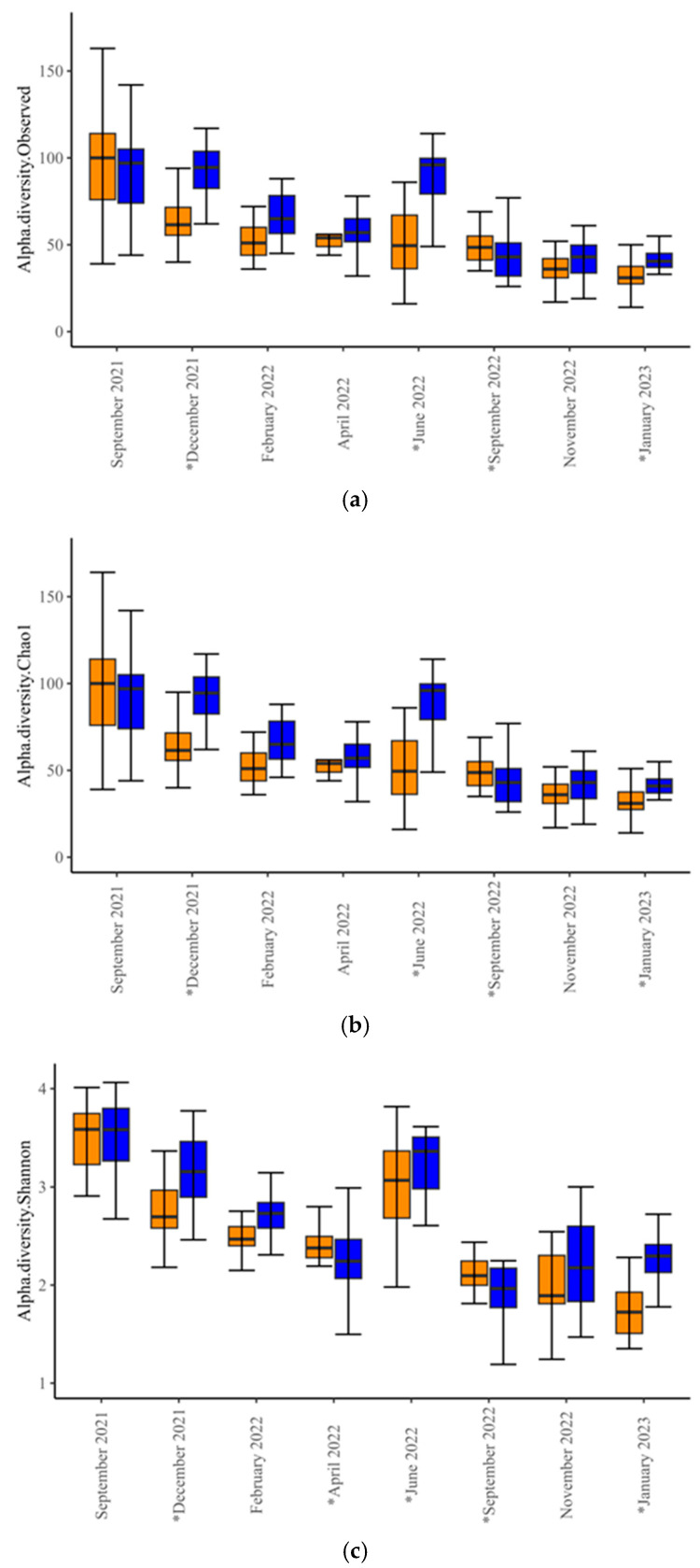
Bacterial alpha diversity analysis (genus level) of control (CON, colored orange,) and supplemented (SUP, colored blue) cows over time. Observed (**a**) and Chao1 (**b**) and Shannon indexes (**c**). Pooled data for overall differences between experimental groups. Observed *p* = 1.2 × 10^−40^, *t*-test = 40.8, Chao1 *p* = 1.9 × 10^−40^, *t*-test = 40.5, Shannon *p* = 2 × 10^−60^, *t*-test = 73.2. Asterisks (*) on the x-axis denote *p* ≤ 0.05 between experimental groups for a given time point. The horizontal bars indicate the median whilst the top and bottom whiskers indicate the upper and lower quartiles respectively.

**Figure 2:**
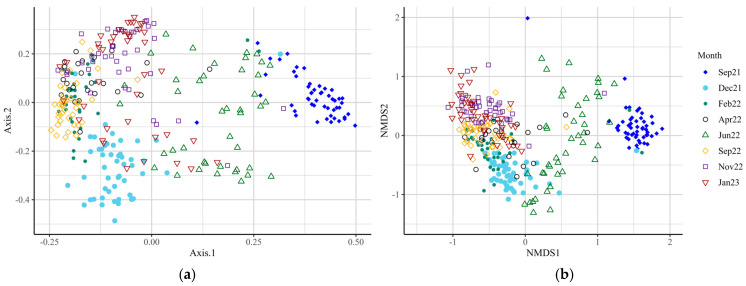
Bacterial beta-diversity (genus level) analysis of milk, using principal coordinate (**a**) and non-metric multi-dimensional scaling (**b**), across eight time points from September 2021 to January 2023. Microbial diversity differed significantly over the 16 months of the trial. F-value = 6.5, R^2^ = 0.02, *p* = 0.001, stress = 0.13.

**Figure 3:**
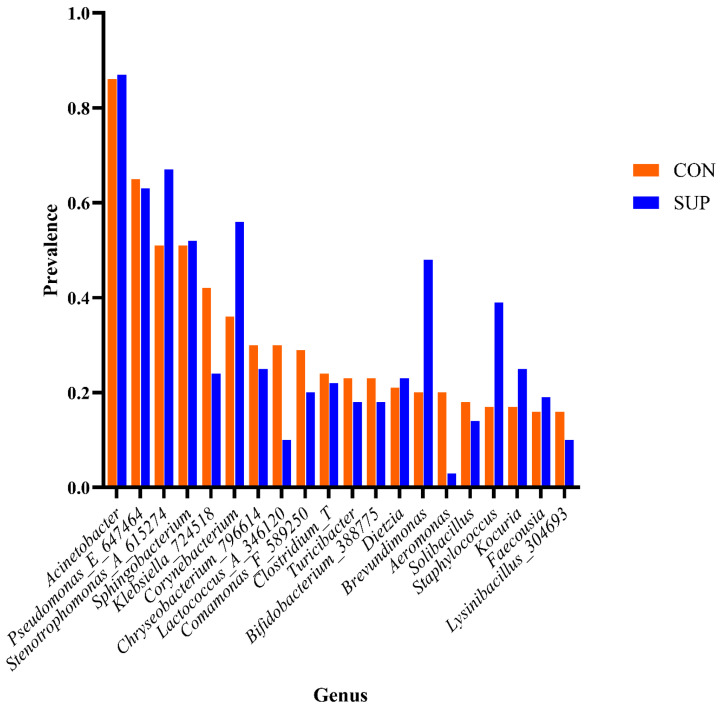
Prevalence of the top twenty genera found in the core microbiota of milk of control (CON) and supplemented (SUP) group cows.

Alpha-diversity analysis revealed differences in microbial diversity within-experimental groups (*p* < 0.001, *t*-test = −3.581 for Observed; *p* < 0.001, *t*-test = −3.546 for Chao1; and *p* = 0.02, *t*-test = −2.3492 for Shannon index). Within-time-points, differences between experimental groups occurred only in December 2021, June and September 2022, and January 2023 (Figure 1, Supplementary Table S1), indicating significant differences in species richness and evenness at these time points. Beta-diversity analysis at the genus level revealed differences in microbial diversity between SUP and CON cows overall (F-value = 6.53; R^2^ = 0.02; *p* = 0.001; Stress = 0.129), and at all time points (Figure 2, Supplementary Table S2, *p* = 0.017–0.001). The core microbiota of both SUP and CON cows was identified, with around 200 genera identified for each group (Supplementary File S1). Of these, 127 were detected in both CON and SUP, and 15 of the 20 most prevalent genera in CON were also the most prevalent in SUP (Figure 3).

**Figure 4:**
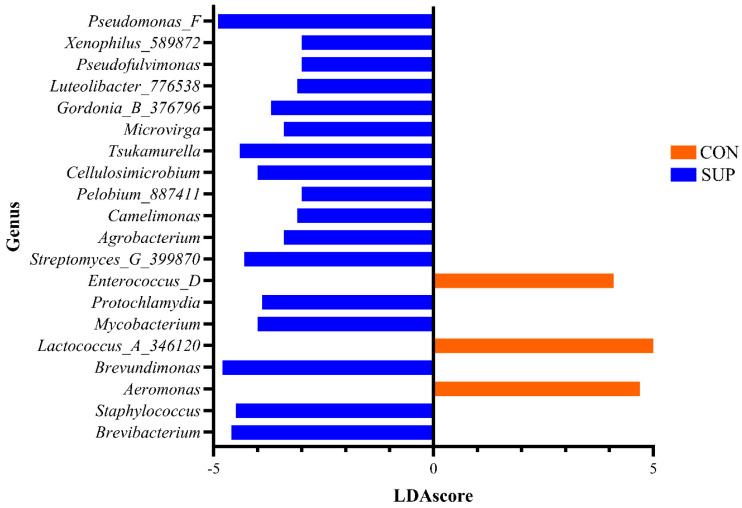
Prevalence of the top twenty genera was associated statistically with differences between the milk of control (CON) and supplemented (SUP) group cows.

Linear discriminant analysis (LDA) effect size (LEfSe) identified 80 genera associated with the differences detected between CON and SUP cows (Supplementary File S1), whilst 340 were associated with the changes over time (Supplementary File S1). The prevalence of the twenty most significant genera which were associated with the differences between the milk microbiota in both groups are shown in Figure 4.

**Figure 5:**
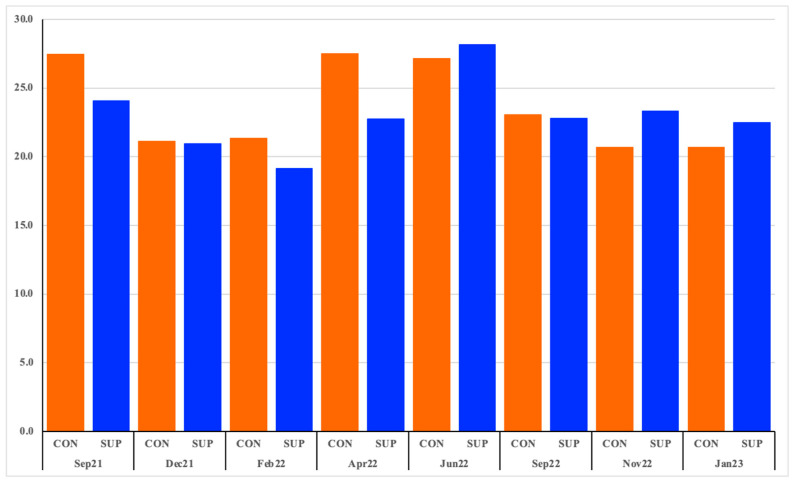
Average milk production (liters per day) of control (CON) and supplemented (SUP) cows over the duration of the experiment.

Each variable was screened with the experimental group in bivariable analysis to determine its overall importance to the data and to ensure there was no overfitting. All variables were then analyzed by multivariable analysis. The top taxa that were significantly associated with the categorical or numerical variables were Age (*Mammaliicoccus*_319276, *Turicibacter*), milk production (*Turicibacter*, *Bifidobacterium*_388775), DIM (*Stenotrophomonas*_A_615274, *Pedobacter*_887417), milk fat percentage (*Pseudomonas*_E_647464, *Lactobacillus*), calendar month (*Jeotgalicoccus*_A_310962, *Planococcus*), milk protein percentage (*Tistrella*, *Pseudomonas*_E_650325), experimental group (*Enterococcus*_B, *Aeromonas*), somatic cell counts (*Paenochrobactrum*, *Pseudochrobactrum*) and trimester of pregnancy (*Dyadobacter*_906144, VFJN01 (Acidimicrobiales)). The total MaAslin2 analysis included 464 genera and 9 variables (Supplementary File S1) with all statistically significant interactions and is provided in Supplementary File S1 and graphically in Figures S2–S5. Correlation analysis of the ratio of Bacillota: Bacteroidota with the percentage of fat in the milk of all cows together found a negative correlation (r = −0.14, *p* = 0.01). No correlation between the Bacillota: Bacteroidota ratio was found between the ruminal and milk data. Ramirez-Garzon et al. [19], wherein the response to the DFM with respect to average, SCC, FatPerc and ProtPerc was reported, found that the DFM had no significant impact on SCC, FatPerc or ProtPerc. The DFM had no significant effect on the variation in SCC in this study (F = 0.375, *p* > 0.05). DFM supplemented cows, however, did produce 0.39 L/day more per cow than the CON group by January 2023. Although there were significant differences in the microbiota from supplementation and over time, there was, however, no statistically significant difference in average milk production at any time point, or overall, between CON and SUP cows (Figure 5).

## 4. Discussion

This study provided the opportunity to investigate the diversity and prevalence of different microbial taxa in the milk of dairy cows over an extended period, in the presence and absence of a DFM, and relate these taxa to key milk productivity data. The milk microbiota changed significantly over time across the 16 months of the study that encompassed all seasons and across two lactations. During the study, both experimental groups were fed PMR, with or without DFM, with ryegrass in the winter and kikuyu in the summer. Perennial ryegrass (*Lolium perenne*) is the most common pasture species used to graze dairy cows in Australiasia because of its long growing season and high nutrient value [44]. Kikuyu is of lower nutritive value, but can survive under marginal climatic conditions, including the Australian summer [44]. These changes in feed composition, quality and availability over the months, and therefore seasons, can influence the milk microbiota, milk quality and quantity by influencing the microbiota of the rumen and subsequent feed efficiency [45,46]. Both grasses significantly impact the ruminal and udder microbiotas, affecting the abundance of genera such as *Prevotella*, *Fibrobacter*, and *Butyrivibrio* in the rumen. These species are important for the degradation of plant material and, therefore, energy production in the cows [47,48]. The differences in nutrient value between the two grasses, along with the microbiota associated with each species, will affect the abundance of various bacterial taxa. This, in turn, will directly influence the ruminal microbiota, digestion, and milk productivity. The udder microbiota will be affected indirectly by the state of the rumen and directly by contact with the microbiotas of the two grasses [47,48]. Those genera that were driving the changes over time in the present study included *Atopostipes*, *Jeotgalicoccus*_A_310962, *Stenotrophomonas*_A_615274, *Planococcus* and *Dietzia*.

The bacterial taxa associated with milk can be classified into those beneficial for food technology, human health applications, spoilage, and those associated with disease (e.g., mastitis) [41]. The present study’s focus, however, was to determine whether DFM supplementation had any impact on milk microbiota. It was shown that DFM supplementation significantly impacted both the species richness and diversity of the microbiota of milk across all time points tested. Those taxa, found by LEfSe analysis, to be driving the differences between CON and SUP cows and are part of the core microbiota, include *Pseudomonas*, *Lactococcus*, and *Staphylococcus*. Many of the taxa that changed significantly over time are also part of the core microbiota, with *Corynebacterium*, *Dietzia*, *Acinetobacter*, and *Bifidobacterium* highly prevalent at the start of the experimental period and *Lactococcus*, *Klebsiella*, *Pseudomonas*, and *Acinetobacter* highly prevalent in the final sampling points of the study. This indicates that taxa associated with the core microbiota drive the changes detected due to both DFM supplementation and over time. Investigation of the association of different taxa with the nine milk productivity measures, analyzed via multivariable analysis, allowed the direct association of taxa with these features. Sixty-four genera were directly associated with experimental groups, fifty of those part of the core microbiota. Given the age of the cows, this association could only remain upon continued supplementation and changes to the microbiota of cows, particularly in mature adults [49]. Weimer et al. [50] exchanged the ruminal fluid from low-efficiency milk-producing cows with ruminal fluid from high-efficiency cows, which then showed an increase in milk production efficiency over seven days. They then reverted to the low-efficiency state and near-original microbiota in around ten days. However, the present study showed that DFM supplementation, whilst having little effect on milk quantity or quality, did significantly influence the microbiota, including core taxa, of cows.

Genera that have previously been found to be associated with differing levels of milk production are *Acinetobacter*, *Actinomyces*, *Bifidobacterium*, *Escherichia-Shigella*, *Fusobacterium*, *Hymenobacter*, *Peptostreptococcus*, *Sphingobacterium* and an unclassified member of the *Ruminococcaceae* [43]. Ninety-eight genera were associated with average milk yield in the present study, including Bifidobacterium, but also *Turicibacter*, *Corynebacterium*, *Stenotrophomonas*_A_615274, *Kaistella*, *Clostridium*_T and *Dietzia*, quite different from that found in other studies. Further investigation is needed to determine what role species associated with each genus play in the amount of milk produced. Nevertheless, DFM supplementation was found in this study to be associated with a numerical increase in milk production [19]. Several studies found a positive correlation between the ratio of Bacillota:Bacteroidota found in the ruminal fluid of dairy cows and the percentage of fat in the milk [34,35,36,37,38]. This ratio has also been associated with amounts of body fat and energy usage in humans and mice [51,52]. In the present study, a significant negative correlation was found between this ratio and the percentage of milk fat. No significant correlation was found between the ruminal and milk ratios in this study. This may be because the rumen is the energy powerhouse of the cow, whilst milk production uses significant amounts of energy to produce milk. Most taxa associated with milk fat percentage belonged to the phylum Bacillota. In agreement with the present study, Xu et al. [6], using a live lactobacilli-based DFM, found no significant influence of the DFM on milk fat percentage. That study used different lactobacilli compared with the present study and a 10-fold lower dose of bacteria over 30 days yet found an average of nine kg more milk than control cows. This is potentially the result of using different species and strains of lactobacilli in the two studies.

The link between the effects of the microbiome and metabolism of the rumen on milk production and quality is well established, yet direct links between the microbiota of the two are yet to be determined [34,53]. Some evidence does suggest interaction between the microbiotas of the gastrointestinal tract and the mammary glands, via the bloodstream, known as the microbiome-gut-mammary axis [53,54]. The milk microbiota data presented herein is part of a larger dataset that includes the fecal and ruminal microbiota analysis corresponding to each milk sample. The DFM had a significant effect on both the fecal and ruminal microbiotas, but not on archaeal populations within the rumen [39,55]. Changes in the ruminal microbiota can induce changes in the udder and fecal microbiota. Whether the changes in milk microbiota are extrinsic, intrinsic or both are difficult to determine from this study. It is possible that metabolic changes in the rumen due to the DFM influenced the milk microbiota and, if the intestinal-udder trafficking of bacteria via the bloodstream is correct, could have influenced the milk microbiota this way.

Somatic cell counts (SCC) have long been used as both an indicator of udder health and a measure of the quality of the milk, with high counts often being associated with poor udder health and milk quality. Milk microbiota associated with low (<200,000 cells/mL), medium (200,000–<800,000 cells/mL) and high (≥800,000 cells/mL) SCC in previous studies included *Staphylococcus* (high SCC), *Marinobacter*, and *Thiopseudomonas* (medium SCC), and eight were associated with low SCC [43]. Unclassified *Bacteroidales* and *Devosia*, *Comomonas* and *Arthrobacter* had significant positive and negative associations with SCC [13]. Oikonomou et al. [56] found two genera positively associated with high SCC, *Sphingobacterium* and *Streptococcus*, and two with low SCC, *Paenibacillus* and *Nocardoides*. The multivariable analysis identified eleven genera positively associated with SCC in the present study. Of these *Cohnella* was associated with low SCC, *Xanothomonas*_A_614439 with medium SCC and *Enterococcus*, was associated with high SCC. How these genera affect SCC is yet to be elucidated. The lactobacilli-based DFM used by Xu et al. [6] significantly reduced the SCC, indicating that DFM supplementation can significantly affect udder health and milk production. In the present study, however, the DFM did not significantly affect the variation in SCC.

Over the cow’s gestation period the levels of protein and fat in the milk increase, particularly in the second and third trimesters, whilst the yield decreases [57,58]. In the present study, multivariable analysis detected thirty-six genera associated with the second and third trimesters of pregnancy. The most significant positive associations were with *Serratia*_A and an unclassified member of the class *Acidimicrobiales* (VFJN01). Significant negative associations were found with *Dyadobacter*_906144, *Mammaliicoccus*_319276 and *Pseudomonas*_F. The biological significance of these associations requires further investigation.

There were a few limitations associated with this study, including the lack of microbiota data for the first nine DIM. Over the first nine days the cows are under considerable metabolic stress and are most at risk of mastitis [59,60]. Microbiota data in this period would be extremely useful for understanding taxa associated with metabolic stress and the development of mastitis. The present study investigated the microbiota of Holstein–Friesian dairy cows in Australia and whilst applicable to similar breeds under similar production systems, would be less comparable to different breeds and productions systems. Whilst multivariable analysis can associate productivity data with the microbiota present the analyses herein are a starting point for fitting a larger model and should be interpreted with care. It would be useful to have a much larger dataset to fit a larger model. The microbial composition of milk found in various studies is highly variable, with findings influenced by sampling techniques, host, and environmental factors [16,40,42]. Whilst the present study focuses on the microbiota of the milk, it is acknowledged that environment and sampling technique can influence the milk microbiota [13,14,15,61]. It is also acknowledged that the methodology used cannot distinguish between live and dead bacteria at the time of sampling but does give a representation of the taxa present [40,41]. Future studies should aim to address the limitations that have been identified, both in the present and in previous studies. Given that many supplementation studies have only been able to change the adult microbiota temporarily, future studies should aim to provide a sub-set of pre-weaned heifers with the supplement and monitor the microbiota through to their first lactation. Both supplemented and control cows could then be sampled for microbiota and monitored for productivity. This could determine the critical point by which to influence cow microbiota for improved productivity. Upon lactation, milk should be sampled directly from the milk cistern [61] and amplified in the presence of propidium monoazide (PMAxx, Biotium, Fremont, CA, USA), which inhibits PCR amplification of DNA from dead bacteria. This would remove many concerns regarding milk sampling contamination and amplify only bacteria that were alive in the sample at the time. Furthermore, metagenomic analysis would be very useful for investigating biochemical potential of the taxa present, to identify associations between microbial biochemical processes and milk production. It could also potentially define what biochemical changes occur due to DFM supplementation and clarify the associations of many of the genera found herein to be associated with milk production and quality. Having this information would not only potentially improve bovine health and productivity but would provide information for new generations of improved DFM supplements.

## 5. Conclusions

Significant differences were found in the microbial diversity within and between cows supplemented with a DFM or left un-supplemented across an extended period. These changes could be attributed to the effects of the direct-fed microbial supplementation and through significant changes in core microbial diversity over time. Taxa driving the changes detected within and between experimental groups were identified. Multivariable analysis identified taxa, including *Paramuribaculum*, an unclassified member of the Ruminococcaceae, *Microvirga*, *Brevilactibacter*, *Providencia*_A_38314, *Cedecea*, *Brumimicrobium*, *Lactobacillus*, *Planococcus*, *Atopostipes*, *Anaerospora*, *Frisingicoccus*, *Aeromonas*, *Protochlamydia*, *Dyadobacter*_906144, an unclassified member of the Acidimicrobiales, *Glutamibacter* and *Lactococcus*_A_346120 that were correlated with experimental group, cow-level factors, and calendar month. Long-term DFM supplementation was associated with changes in milk microbiota composition in Holstein–Friesian dairy cows, impacting microbial communities and milk productivity.

## Data Availability

The raw sequence data obtained for this study is available on NCBI at accession number PRJNA1257071.

## Supplementary Materials

The following supporting information can be downloaded at: https://www.mdpi.com/article/10.3390/ani15142124/s1, Figure S1: Relative abundance of bacterial phyla found in the milk of cows with (SUP) and without (CON) DFM supplementation over the course of the 16-month study. Table S1: Alpha-diversity analysis (genus level) of within-treatment diversity of bacterial communities in milk from CON and SUP cows over the time points tested. Asterisks (*) indicate *p*-values that are statistically significant (*p* ≤ 0.05). Table S2: Beta-diversity analysis (genus level) of the microbial diversity of milk from CON and SUP cows over the eight sampling time points. Asterisks (*) indicate *p*-values that are statistically significant (*p* ≤ 0.05). Figure S2: Heatmap of genera that are significantly associated with Age (years), Average milk (l/day), days in milk (DIM), percentage of fat (FatPerc) and protein (ProtPerc), calendar month (Month), somatic cell count (SCC), experimental group (SUP) and trimester of pregnancy (Trimester). Significant interactions (*p* ≤ 0.05, FDR < 0.2) are colored in different shadings of blue, with the most intense being the most significant. Genera are in reverse alphabetical order. Figure S3: Heatmap of genera that are significantly associated with Age (years), Average milk (l/day), days in milk (DIM), percentage of fat (FatPerc) and protein (ProtPerc), calendar month (Month), somatic cell count (SCC), experimental group (SUP) and trimester of pregnancy (Trimester). Significant interactions (*p* ≤ 0.05, FDR < 0.2) are colored in different shadings of red, with the most intense being the most significant. Genera are in reverse alphabetical order. Figure S4: Heatmap of genera that are significantly associated with Age (years), Average milk (l/day), days in milk (DIM), percentage of fat (FatPerc) and protein (ProtPerc), calendar month (Month), somatic cell count (SCC), experimental group (SUP) and trimester of pregnancy (Trimester). Significant interactions (*p* ≤ 0.05, FDR < 0.2) are colored in different shadings of red, with the most intense being the most significant. Genera are in reverse alphabetical order. Figure S5: Heatmap of genera that are significantly associated with Age (years), Average milk (l/day), days in milk (DIM), percentage of fat (FatPerc) and protein (ProtPerc), calendar month (Month), somatic cell count (SCC), experimental group (SUP) and trimester of pregnancy (Trimester). Significant interactions (*p* ≤ 0.05, FDR < 0.2) are colored in different shadings of red, with the most intense being the most significant. Genera are in reverse alphabetical order. File S2: Data for classification to phylum, family and genus.

## Abbreviations

The following abbreviations are used in this manuscript:

DFM	Direct-fed microbial
SUP	Supplemented with the DFM
CON	Control group
PMR	Partial mixed ration
RMIT	Royal Melbourne Institute of Technology
PCR	Polymerase chain reaction
ASV	Amplified sequence variant
MA	Microbiome Analyst
NMDS	Non-metric multidimensional scaling
PCoA	Principal coordinate analysis
DIM	Days in milk
SCC	Somatic cell counts
FatPerc	Percentage of fat in the milk
ProtPerc	Percentage of protein in the milk

## References

[1] Wu Y., Wang L., Luo R., Chen H., Nie C., Niu J., Chen C., Xu Y., Li X., Zhang W. (2021). Effect of a Multispecies Probiotic Mixture on the Growth and Incidence of Diarrhea, Immune Function, and Fecal Microbiota of Pre-weaning Dairy Calves. Front. Microbiol..

[2] Wang L., Sun H., Gao H., Xia Y., Zan L., Zhao C. (2023). A meta-analysis on the effects of probiotics on the performance of pre-weaning dairy calves. J. Anim. Sci. Biotechnol..

[3] El Jeni R., Villot C., Koyun O.Y., Osorio-Doblado A., Baloyi J.J., Lourenco J.M., Steele M., Callaway T.R. (2024). Invited review “Probiotic” approaches to improving dairy production Reassessing “magic foo-foo dust”. J. Dairy Sci..

[4] Hernández J., Benedito J.L., Abuelo A., Castillo C. (2014). Ruminal Acidosis in Feedlot: From Aetiology to Prevention. Sci. World J..

[5] Renaud D.L., Kelton D.F., Weese J.S., Noble C., Duffield T.F. (2019). Evaluation of a multispecies probiotic as a supportive treatment for diarrhea in dairy calves: A randomized clinical trial. J. Dairy Sci..

[6] Xu H., Huang W., Hou Q., Kwok L., Sun Z., Ma H., Zhao F., Lee Y.-K., Zhang H. (2017). The effects of probiotics administration on the milk production, milk components and fecal bacteria microbiota of dairy cows. Sci. Bull..

[7] Nalla K., Manda N.K., Dhillon H.S., Kanade S.R., Rokana N., Hess M., Puniva A.K. (2022). Impact of Probiotics on Dairy Production Efficiency. Front. Microbiol..

[8] Elghandour M.M.Y., Salem A.Z.M., Martínez-Castañeda J.S., Camacho L.M., Kholif A.E., Vázquez-Chagoyán J.C. (2015). Direct-fed microbes: A tool for improving the utilization of low quality roughages in ruminants. J. Int. Agric..

[9] Izhar M.Z., Nawaz M., Yaqub T., Avais M. (2025). Effect of probiotic *Lactobacillus plantarum* CM49 on microbial profile and lactobacilli counts in milk of mastitic cattle. BMC Microbiol..

[10] Kim I.S., Hur Y.K., Kim E.J., Ahn Y.-T., Kim J.G., Choi Y.-J., Huh C.S. (2017). Comparative analysis of the microbial communities in raw milk produced in different regions of Korea. Asian-Australas. J. Anim. Sci..

[11] Porcellato D., Meisal R., Bombelli A., Narvhus J.A. (2020). A core microbiota dominates a rich microbial diversity in the bovine udder and may indicate presence of dysbiosis. Sci. Rep..

[12] Ryu S., Park W.S., Yun B., Shin M., Go G., Kim J.N., Oh S., Kim Y. (2021). Diversity and characteristics of raw milk microbiota from Korean dairy farms using metagenomic and culturomic analysis. Food Control.

[13] Derakhshani H., Fehr K.B., Sepehri S., Francoz D., De Buck J., Barkema H.W., Plaizier J.C., Khafipour E. (2018). Invited review: Microbiota of the bovine udder Contributing factors and potential implications for udder health and mastitis susceptibility. J. Dairy Sci..

[14] Derakhshani H., Plaizier J.C., De Buck J., Barkema H.W., Khafipour E. (2018). Composition of the teat canal and intramammary microbiota of dairy cows subjected to antimicrobial dry cow therapy and internal teat sealant. J. Dairy Sci..

[15] Metzger S.A., Hernandez L.L., Suen G., Ruegg P.L. (2018). Understanding the Milk Microbiota. Vet. Clin. N. Am. Food Anim. Pract..

[16] Ruegg P.L. (2022). The bovine milk microbiome—An evolving science. Domest. Anim. Endocrinol..

[17] Derakhshani H., Plaizier J.C., De Buck J., Barkema H.W., Khafipour E. (2020). Composition and co-occurrence patterns of the microbiota of different niches of the bovine mammary gland: Potential associations with mastitis susceptibility, udder inflammation, and teat-end hyperkeratosis. Anim. Microbiome.

[18] Zigo F., Vasil’ M., Ondrašovičová S., Výrostková J., Bujok J., Pecka-Kielb E. (2021). Maintaining Optimal Mammary Gland Health and Prevention of Mastitis. Front. Vet. Sci..

[19] Ramirez-Garzon O., Al-Alawneh J.I., Barber D., Liu H., Soust M. (2024). The Effect of a Direct Fed Microbial on Liveweight and Milk Production in Dairy Cattle. Animals.

[20] Campbell B.E., Van T.T.H., Ramsland P.A., Elbourne A., Gurtler V. (2024). Using next generation sequencing to study host-pathogen interactions. Methods in Microbiology Series, Microbes at Bio/Nano Interfaces.

[21] Takahashi S., Tomita J., Nishioka K., Hisada T., Nishijima M. (2014). Development of a Prokaryotic Universal Primer for Simultaneous Analysis of Bacteria and Archaea Using Next-Generation Sequencing. PLoS ONE.

[22] Gohl D.M., Vangay P., Garbe J., MacLean A., Hauge A., Becker A., Gould T.J., Clayton J.B., Johnson T.J., Hunter R. (2016). Systematic improvement of amplicon marker gene methods for increased accuracy in microbiome studies. Nat. Biotechnol..

[23] Bolyen E., Rideout J.R., Dillon M.R., Bokulich N.A., Abnet C.C., Al-Ghalith G.A., Alexander H., Alm E.J., Arumugam M., Asnicar F. (2019). Reproducible, interactive, scalable and extensible microbiome data science using QIIME 2. Nat. Biotechnol..

[24] Callahan B.J., McMurdie P.J., Rosen M.J., Han A.W., Johnson A.J.A., Holmes S.P. (2016). DADA2 High-resolution sample inference from Illumina amplicon data. Nat. Methods.

[25] McDonald D., Jiang Y., Balaban M., Cantrell K., Zhu Q., Gonzalez A., Morton J.T., Nicolaou G., Parks D.H., Karst S.M. (2023). Greengenes2 unifies microbial data in a single reference tree. Nat. Biotechnol..

[26] Chao A. (1984). Nonparametric Estimation of the Number of Classes in a Population. Scand. J. Stat..

[27] Shannon C., Weaver W. (1949). The Mathematical Theory of Communication.

[28] Rabinowitz G.B. (1975). An Introduction to Nonmetric Multidimensional Scaling. Am. J. Polit. Sci..

[29] Gower J.C. (1966). Some distance properties of latent root and vector methods used in multivariate analysis. Biometrika.

[30] Mallick H., Rahnavard A., McIver L.J., Ma S., Zhang Y., Nguyen L.H., Tickle T.L., Weingart G., Ren B., Schwager E.H. (2021). Multivariable association discovery in population-scale meta-omics studies. PLoS Comput. Biol..

[31] Dahan E., Martin V.M., Yassour M. (2022). EasyMap—An Interactive Web Tool for Evaluating and Comparing Associations of Clinical Variables and Microbiome Composition. Front. Cell Infect. Microbiol..

[32] Wickham H. (2016). ggplot2 Elegant Graphics for Data Analysis.

[33] RStudio Team (2020). RStudio Integrated Development for R.

[34] Jami E., White B.A., Mizrahi I. (2014). Potential Role of the Bovine Rumen Microbiome in Modulating Milk Composition and Feed Efficiency. PLoS ONE.

[35] Schären M., Frahm J., Kersten S., Meyer U., Hummel J., Breves G., Dänicke S. (2018). Interrelations between the rumen microbiota and production, behavioral, rumen fermentation, metabolic, and immunological attributes of dairy cows. J. Dairy Sci..

[36] Si B., Liu K., Huang G., Chen M., Yang J., Wu X., Li N., Tang W., Zhao S., Zheng N. (2023). Relationship between rumen bacterial community and milk fat in dairy cows. Front. Microbiol..

[37] Hassan F., Ebeid H.M., Tang Z., Li M., Peng L., Peng K., Liang X., Yang C. (2020). A Mixed Phytogenic Modulates the Rumen Bacteria Composition and Milk Fatty Acid Profile of Water Buffaloes. Front. Vet. Sci..

[38] Keum G.B., Pandey S., Kim E.S., Doo H., Kwak J., Ryu S., Choi Y., Kang J., Kim S., Kim H.B. (2024). Understanding the Diversity and Roles of the Ruminal Microbiome. J. Microbiol..

[39] Campbell B.E., Hassan M.M., Moore R.J., Olchowy T., Soust M., Al Jassim R., Alawneh J.I. (2025). Temporal changes in ruminal microbiota composition and diversity in dairy cows supplemented with a lactobacilli-based DFM. Front. Vet. Sci..

[40] Oikonomou G., Addis M.F., Chassard C., Nader-Macias M.E.F., Grant I., Delbès C., Bogni C.I., Le Loir Y., Even S. (2020). Milk Microbiota: What Are We Exactly Talking About?. Front. Microbiol..

[41] Quigley L., O’Sullivan O., Stanton C., Beresford T.P., Ross R.P., Fitzgerald G.F., Cotter P.D. (2013). The complex microbiota of raw milk. FEMS Microbiol. Rev..

[42] Machado S.G., Baglinière F., Marchand S., Van Coillie E., Vanetti M.C.D., De Block J., Heyndrickx M. (2017). The Biodiversity of the Microbiota Producing Heat-Resistant Enzymes Responsible for Spoilage in Processed Bovine Milk and Dairy Products. Front. Microbiol..

[43] Williamson J.R., Callaway T.R., Lourenco J.M., Ryman V.E. (2022). Characterization of rumen, fecal, and milk microbiota in lactating dairy cows. Front. Microbiol..

[44] Fulkerson W.J., Neal J.S., Clark C.F., Horadagoda A., Nandra K.S., Barchia I. (2007). Nutritive value of forage species grown in the warm temperate climate of Australia for dairy cows Grasses and legumes. Livest. Sci..

[45] Li N., Wang Y., You C., Ren J., Chen W., Zheng H., Liu Z. (2018). Variation in Raw Milk Microbiota Throughout 12 Months and the Impact of Weather Conditions. Sci. Rep..

[46] Magan J.B., O’Callaghan T.F., Kelly A.L., McCarthy N.A. (2021). Compositional and functional properties of milk and dairy products derived from cows fed pasture or concentrate-based diets. Comp. Rev. Food Sci. Food Safe.

[47] Huws S.A., Edwards J.E., Creevey C.J., Stevens P.R., Lin W., Girdwood S.E., Pachebat J.A., Kingston-Smith A.H. (2016). Temporal dynamics of the metabolically active rumen bacteria colonizing fresh perennial ryegrass. FEMS Microbiol. Ecol..

[48] Huws S.A., Edwards J.E., Lin W., Rubino F., Alston M., Swarbreck D., Caim S., Stevens P.R., Pachebat J., Won M.-Y. (2021). Microbiomes attached to fresh perennial ryegrass are temporally resilient and adapt to changing ecological niches. Microbiome.

[49] Clemmons B.A., Voy B.H., Myer P.R. (2019). Altering the Gut Microbiome of Cattle Considerations of Host-Microbiome Interactions for Persistent Microbiome Manipulation. Microb. Ecol..

[50] Weimer P.J., Cox M.S., De Paula T.V., Lin M., Hall M.B., Suen G. (2017). Transient changes in milk production efficiency and bacterial community composition resulting from near-total exchange of ruminal contents between high- and low-efficiency Holstein cows. J. Dairy Sci..

[51] Ley R.E., Turnbaugh P.J., Klein S., Gordon J.I. (2006). Microbial ecology human gut microbes associated with obesity. Nature.

[52] Turnbaugh P.J., Ley R.E., Mahowald M.A., Magrini V., Mardis E.R., Gordon J.I. (2006). An obesity-associated gut microbiome with increased capacity for energy harvest. Nature.

[53] Welch C.B., Ryman V.E., Pringle T.D., Lourenco J.M. (2022). Utilizing the Gastrointestinal Microbiota to Modulate Cattle Health through the Microbiome-Gut-Organ Axes. Microorganisms.

[54] Young W., Hine B.C., Wallace O.A.M., Callaghan M., Bibiloni R. (2015). Transfer of intestinal bacterial components to mammary secretions in the cow. Peer J..

[55] Campbell B.E., Hassan M.M., Moore R.J., Olchowy T., Ranjbar S., Soust M., Ramirez-Garzon O., Al Jassim R., Alawneh J.I. (2024). Temporal Changes in Faecal Microbiota Composition and Diversity in Dairy Cows Supplemented with a Lactobacillus-Based Direct-Fed Microbial. Animals.

[56] Oikonomou G., Bicalho M.L., Meira E., Rossi R.E., Foditsch C., Machado V.S., Teixeira A.G.V., Santisteban C., Schukken Y.H., Bicalho R.C. (2014). Microbiota of Cow’s Milk; Distinguishing Healthy, Sub-Clinically and Clinically Diseased Quarters. PLoS ONE.

[57] Loker S., Miglior F., Bohmanova J., Jamrozik J., Schaeffer L.R. (2009). Phenotypic analysis of pregnancy effect on milk, fat, and protein yields of Canadian Ayrshire, Jersey, Brown Swiss, and Guernsey breeds. J. Dairy Sci..

[58] Penasa M., De Marchi M., Cassandro M. (2016). Short communication Effects of pregnancy on milk yield, composition traits, and coagulation properties of Holstein cows. J. Dairy Sci..

[59] Mallard B.A., Dekkers J.C., Ireland M.J., Leslie K.E., Sharif S., Vankampen C.L., Wagter L., Wilkie B.N. (1998). Alteration in Immune Responsiveness During the Peripartum Period and Its Ramification on Dairy Cow and Calf Health. J. Dairy Sci..

[60] Kessel S., Stroehl M., Meyer H.H.D., Hiss S., Sauerwein H., Schwarz F.J., Bruckmaier R.M. (2008). Individual variability in physiological adaptation to metabolic stress during early lactation in dairy cows kept under equal conditions. J. Anim. Sci..

[61] Dahlberg J., Williams J.E., McGuire M.A., Peterson H.K., Östensson K., Agenäs S., Dicksved J., Waller K.P. (2020). Microbiota of bovine milk, teat skin, and teat canal: Similarity and variation due to sampling technique and milk fraction. J. Dairy Sci..

