# Supplementary material for: Analysis of the Microbiota of Milk from Holstein–Friesian Dairy Cows Fed a Microbial Supplement
Source: Animals (Basel). 2025 Jul 18;15(14):2124. doi: 10.3390/ani15142124 (PMC12291751; doi:10.3390/ani15142124)
Supplement: Supplementary file 1 [file animals-15-02124-s001.zip › Supplementary_file_S1.pdf]

Supplemental Figures and Tables

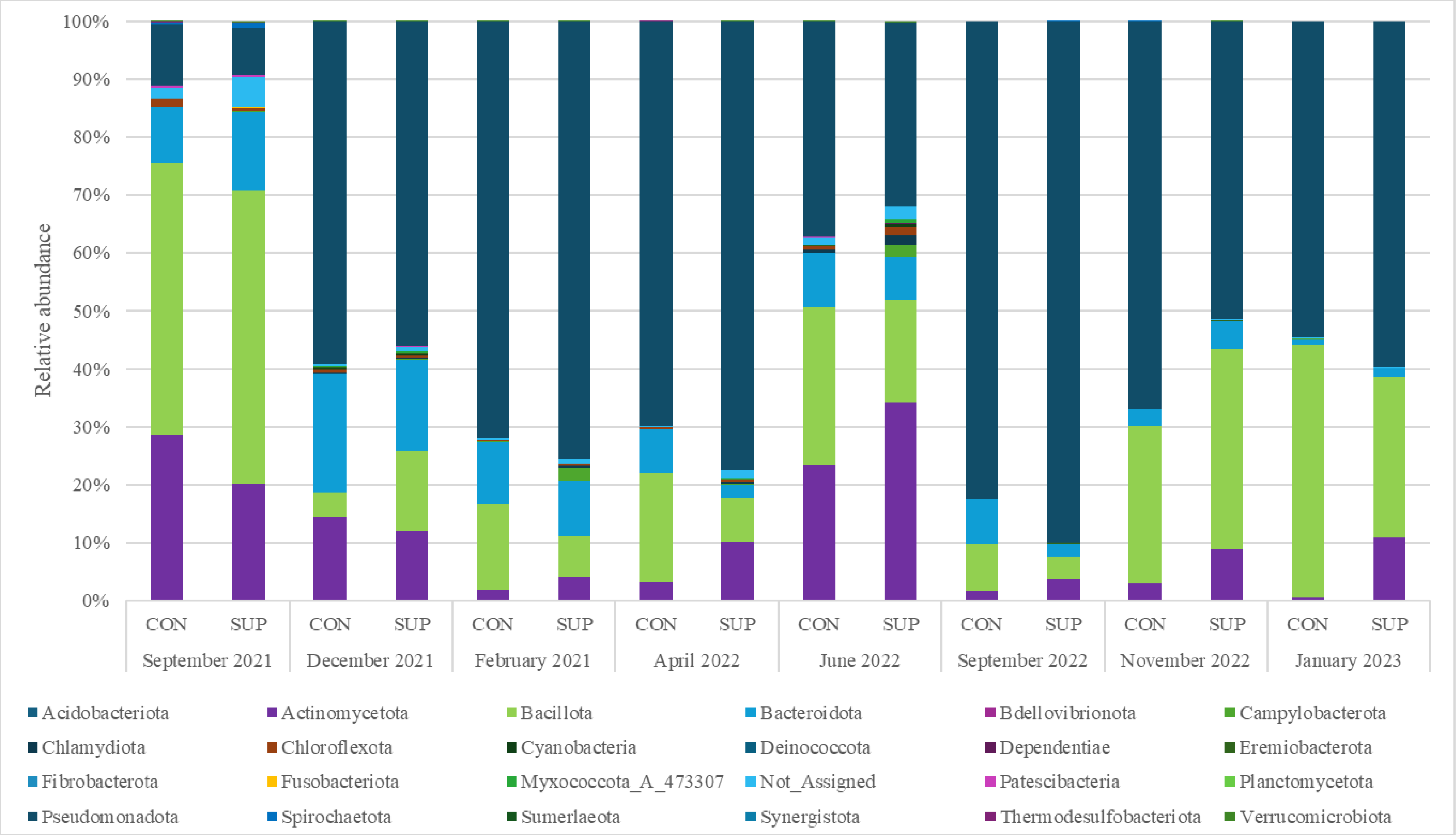

Figure S1: Relative abundance of bacterial phyla found in the milk of cows with (SUP) and without (CON) DFM supplementation over the course of the 16-month study.

Table S1: Alpha-diversity analysis (genus level) of within-treatment diversity of bacterial communities in milk from CON and SUP cows over the time points tested. Asterisks (*) indicate p-values that are statistically significant (P ≤ 0.05).

	**Observed**		**Chao1**		**Shannon**	
	**p-value**	**T-test**	**p-value**	**T-test**	**p-value**	**T-test**
September 2021	0.09	-1.7	0.09	-1.7	0.59	-0.5
December 2021	5.9E-06*	5.3	5.7E-06*	5.3	0.002*	3.4
February 2022	0.2	1.3	0.2	1.3	0.2	1.3
April 2022	0.7	-0.4	0.7	-0.4	0.02*	-2.5
June 2022	5.0E-08*	6.7	5.2E-08*	6.7	0.02*	2.3
September 2022	0.001*	-3.5	0.001*	-3.5	0.009*	-2.7
November 2022	0.4	0.9	0.4	0.9	0.2	1.4
January 2023	0.01*	2.6	0.01*	2.7	2.1E-06*	5.7

Table S2: Beta-diversity analysis (genus level) of the microbial diversity of milk from CON and SUP cows over the eight sampling time points. Asterisks (*) indicate p-values that are statistically significant (P ≤ 0.05).

	**F-value**	**R^2**	**p-value**	**Stress**
September 2021	3.8	0.07	0.001*	4.1563E-05
December 2021	4.4	0.08	0.001*	3.7006E-05
February 2022	6.1	0.2	0.001*	5.0684E-05
April 2022	3.7	0.1	0.001*	0.18
June 2022	7.6	0.2	0.001*	0.15
September 2022	9.5	0.2	0.001*	0.22
November 2022	2.4	0.6	0.017-0.02*	0.17
January 2023	23.4	0.4	0.001*	0.14

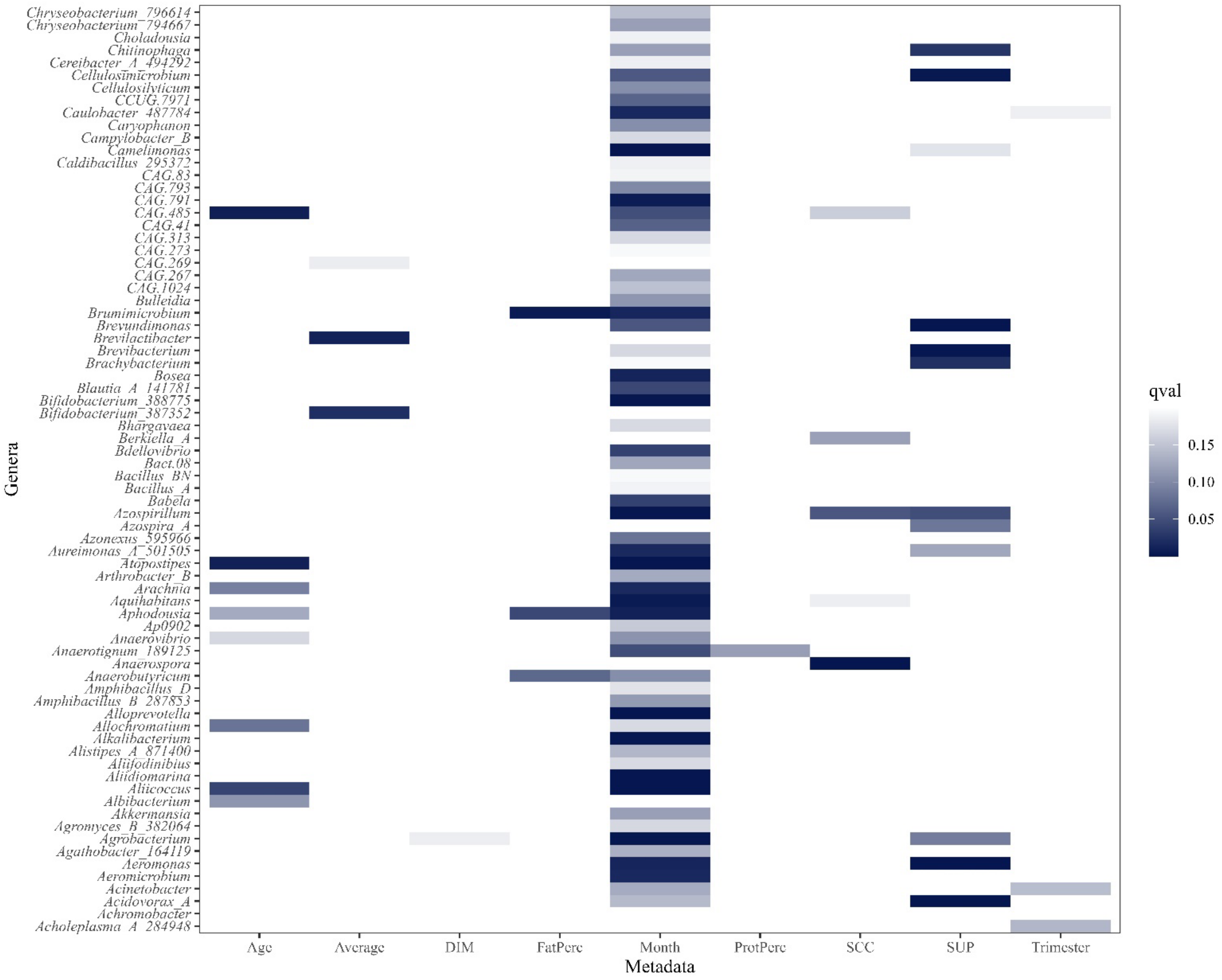

Figure S2: Heatmap of genera that are significantly associated with Age (years), Average milk (l/day), days in milk (DIM), percentage of fat (FatPerc) and protein (ProtPerc), calendar month (Month), somatic cell count (SCC), experimental group (SUP) and trimester of pregnancy (Trimester). Significant interactions (P ≤ 0.05, FDR < 0.2) are colored in different shadings of blue, with the most intense being the most significant. Genera are in reverse alphabetical order.

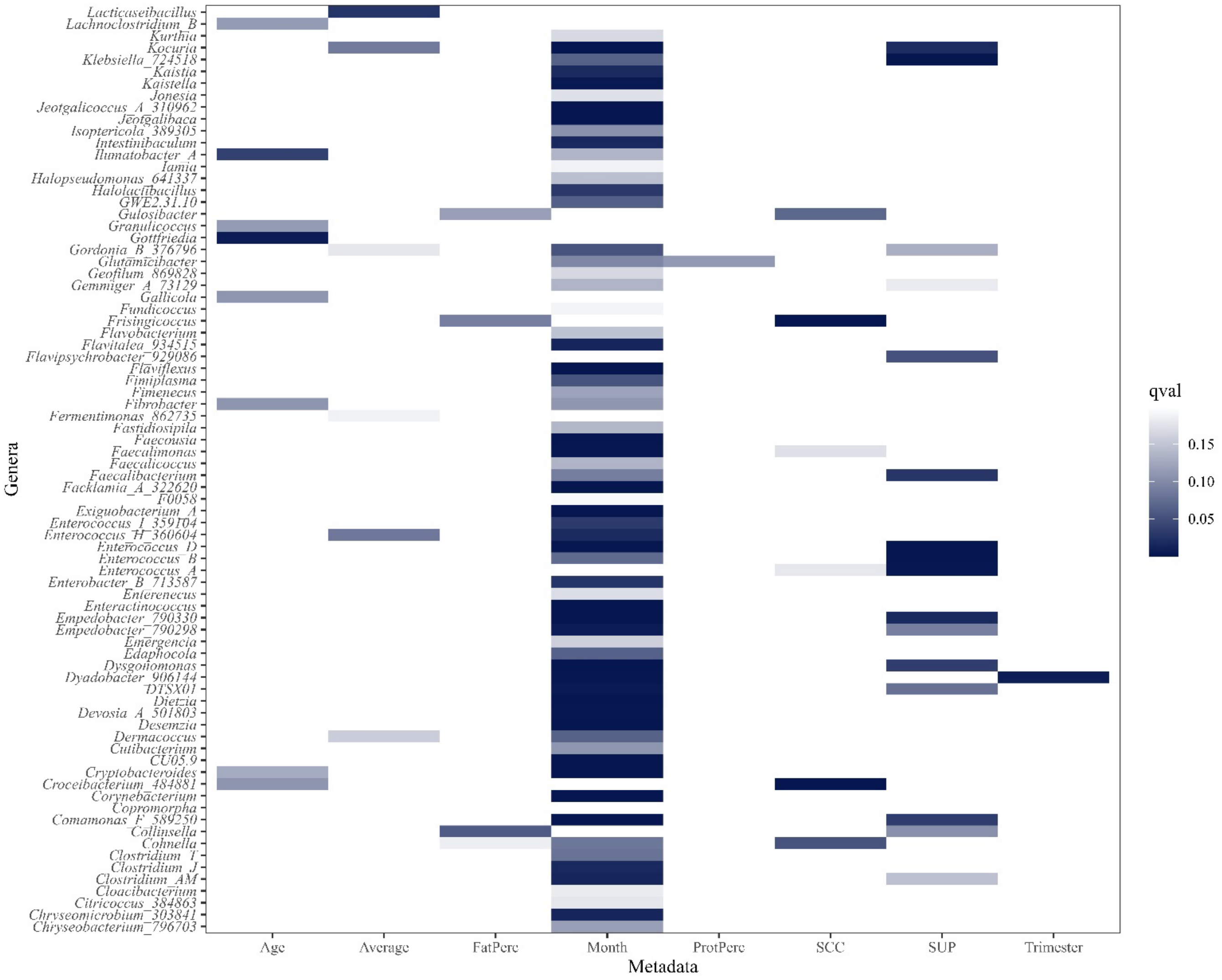

Figure S3: Heatmap of genera that are significantly associated with Age (years), Average milk (l/day), days in milk (DIM), percentage of fat (FatPerc) and protein (ProtPerc), calendar month (Month), somatic cell count (SCC), experimental group (SUP) and trimester of pregnancy (Trimester). Significant interactions (P ≤ 0.05, FDR < 0.2) are colored in different shadings of red, with the most intense being the most significant. Genera are in reverse alphabetical order.

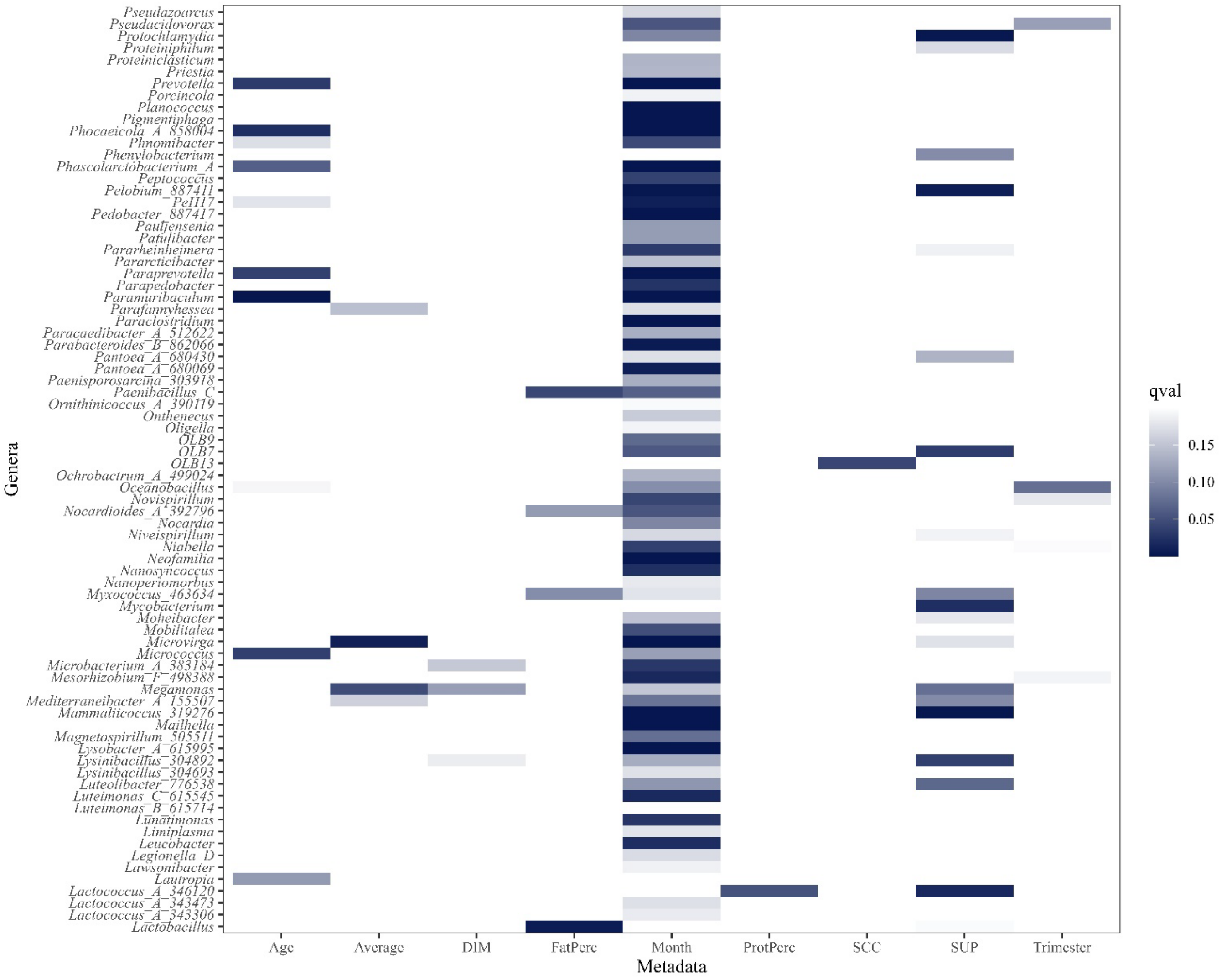

Figure S4: Heatmap of genera that are significantly associated with Age (years), Average milk (l/day), days in milk (DIM), percentage of fat (FatPerc) and protein (ProtPerc), calendar month (Month), somatic cell count (SCC), experimental group (SUP) and trimester of pregnancy (Trimester). Significant interactions ($P \leq 0.05$, FDR < 0.2) are colored in different shadings of red, with the most intense being the most significant. Genera are in reverse alphabetical order.

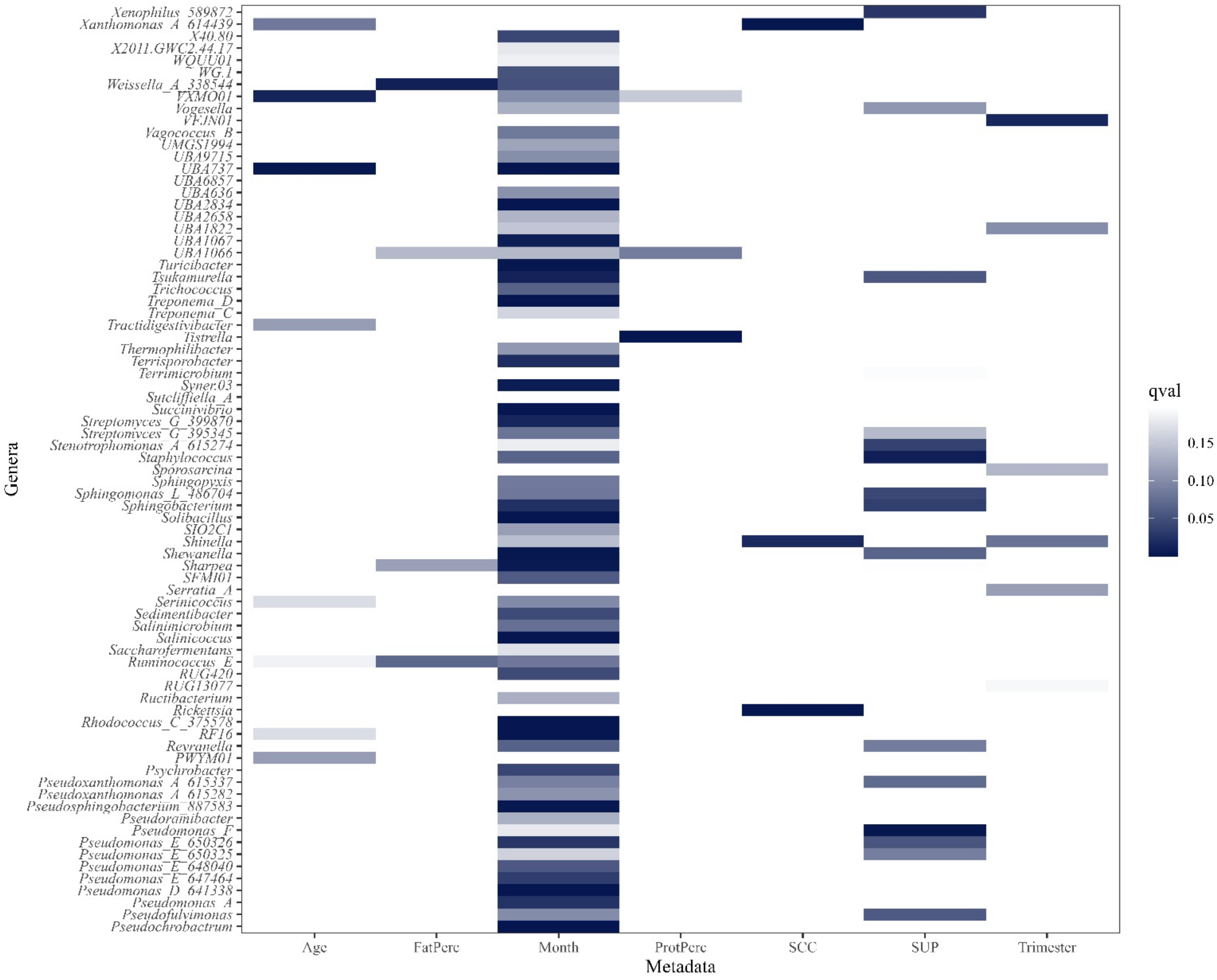

Figure S5: Heatmap of genera that are significantly associated with Age (years), Average milk (l/day), days in milk (DIM), percentage of fat (FatPerc) and protein (ProtPerc), calendar month (Month), somatic cell count (SCC), experimental group (SUP) and trimester of pregnancy (Trimester). Significant interactions ($P \leq 0.05$, $FDR < 0.2$) are colored in different shadings of red, with the most intense being the most significant. Genera are in reverse alphabetical order.
